# Fracture Toughness of Ordinary Plain Concrete Under Three-Point Bending Based on Double-K and Boundary Effect Fracture Models

**DOI:** 10.3390/ma17215387

**Published:** 2024-11-04

**Authors:** Huating Chen, Yifan Zhuo, Dewang Li, Yan Huang

**Affiliations:** 1 State Key Laboratory of Bridge Safety and Resilience, Beijing University of Technology, Beijing 100124, China; 2 Department of Civil Engineering, Beijing University of Technology, Beijing 100124, China; zhuoyf@emails.bjut.edu.cn; 3 Guangzhou Highway Co., Ltd., Guangzhou 510555, China; lidewang.gjtjt@gmail.com

**Keywords:** fracture test, single-edge notched beam, ordinary concrete, fracture toughness, double-K fracture model, boundary effect model

## Abstract

Fracture tests are a necessary means to obtain the fracture properties of concrete, which are crucial material parameters for the fracture analysis of concrete structures. This study aims to fill the gap of insufficient test results on the fracture toughness of widely used ordinary C40~C60 concrete. A three-point bending fracture test was conducted on 28 plain concrete and 6 reinforced concrete single-edge notched beam specimens with various depths of prefabricated notches. The results are reported, including the failure pattern, crack initiation load, peak load, and complete load versus crack mouth opening displacement curves. The cracking load showed significant variation due to differences in notch prefabrication and aggregate distribution, while the peak load decreased nonlinearly with an increase in the notch-to-height ratio. The reinforced concrete beams showed a significantly higher peak load than the plain concrete beams, attributed to the restraint of steel reinforcement, but the measured cracking load was comparable. A compliance versus notch-to-height ratio curve was derived for future applications, such as estimating crack length in crack growth rate tests. Finally, fracture toughness was determined based on the double-K fracture model and the boundary effect model. The average fracture toughness value for C50 concrete from this study was 2.0 $MPa\cdot \sqrt{m}$, slightly smaller than that of lower-strength concrete, indicating the strength and ductility dependency of concrete fracture toughness. The fracture toughness calculated from the two models is consistent, and both methods employ a closed-form solution and are practical to use. The derived fracture toughness was insensitive to the discrete parameters in the boundary effect model. The insights gained from this study significantly contribute to our understanding of the fracture toughness properties of ordinary structural concrete, highlighting its potential to shape future studies and applications in the field.

## 1. Introduction

Understanding the fracture behavior of concrete structures, particularly bridge decks, is critical to ensuring their structural safety and integrity [1]. The development of macroscopic cracks under service conditions is a phenomenon that has attracted significant attention in engineering and academic communities [2,3]. Unlike structural steels, concrete exhibits a quasi-brittle nonlinear behavior due to the fracture process zone (FPZ) [2,4]. This zone is characterized by numerous heterogeneous micro-crackings, or fictitious cracks, near the macroscopic crack tip [2,5]. These fictitious cracks are assumed to be able to transfer stress, and the phenomenon of concrete fracture must be explained by considering such crack bridging stress or cohesive stress [2,6].

Fracture analysis of concrete structures involves evaluating the structural safety of the structure containing certain cracks and determining the maximum crack size that can be tolerated. Such analysis involves crack driving force on the one hand and crack resistance as basic material parameters on the other hand. The driving force regarding stress intensity factor or energy release rate is analyzed from nonlinear fracture mechanics with due consideration for fictitious crack [7]. The resistance relies on concrete’s fracture properties, the critical values beyond which unstable fracture occurs. These fracture parameter indices include fracture toughness [8], fracture energy [9,10], and critical crack tip opening displacement [11]. Numerous research studies have demonstrated that these three fracture properties are related and exchangeable [12]. Fracture toughness, a critical stress intensity factor value in concrete fracture mechanics that indicates concrete’s ability to resist crack propagation, is the most widely researched fracture parameter [12,13].

The fracture toughness of concrete is primarily obtained through fracture tests of small-scale notched specimens. Various types of specimens, including bending beams and splitting wedges, have been used [11,14]. Because of savings in material and simple operation for testing, single-edge notched beam (SENB) specimens loaded in three-point bending is the most commonly used specimen, especially for flexural applications [8]. Scholars have conducted numerous experimental studies on concrete fractures since the 1980s [15,16]. The effects of many relevant parameters, such as mix ratio, notch-to-height ratio, specimen size, loading rate, fatigue loading, and low temperature, on fracture toughness have been investigated [17,18,19,20,21,22]. In recent years, the primary focus of research on concrete fracture toughness has shifted towards self-compacting concrete [18,23] and the effects of coral aggregates [24], recycled aggregates [25], fiber reinforcement [26,27], and lightweight aggregates [28]. Existing works have mainly been conducted on low-strength ordinary concrete traditionally and recently on innovative high-strength composite concrete. Nevertheless, the research on normal-grade ordinary concrete commonly used in bridge engineering, say, C40~C60, is limited.

It should be noted that fracture toughness is not a physical quantity that can be measured directly from experiments. It is calculated indirectly from some equations based on nonlinear fracture mechanics. The crucial parameter is the amount of fictitious crack extension or the size of the fracture process zone [2,4]. In case optical or visual measurement methods are not feasible, the calibrated compliance method is the primary method for indirect crack length measurement. This method requires applying unloading near maximum load and deduces the sub-critical crack extension from the difference between loading and unloading compliance [8,29]. However, an unloading process shortly before or after the peak load is challenging because plain concrete specimens tend to break abruptly once reaching the peak load.

Various fracture models have been proposed to determine fracture toughness from SENB specimens without the need for unloading at peak load, among which is the double-K fracture model (DKFM), the most widely used and conventional method. This model utilizes two fracture toughness values to divide crack extension into three distinctive stages and explicitly recognize a small amount of fictitious crack extension before unstable fracture [30]. DKFM could calculate the unstable fracture toughness directly from peak load and the corresponding crack mouth opening displacement based on the principle of linear asymptotic superposition [30,31].

Another fracture model, the boundary effect model (BEM), has attracted considerable research attention over recent years. The model’s underlying assumptions are that the tensile strength criterion applies if the crack size, considering the effect of specimen boundaries, is extremely small, the fracture toughness criterion applies if the crack size, considering boundary effect, is sufficiently large, and the transitional quasi-brittle fracture, occurring for cases in between, is asymptotic to the strength-controlled or fracture toughness-controlled limits [4,9]. The model was initially formulated to tackle the size effect problem and required curve fitting [4,32]. Recently, a closed-form solution of BEM has been proposed to deduce fracture toughness directly from the peak load of the fracture test [22,33]. This method is promising and has been successfully applied to quasi-brittle materials such as granite [34], sandstone [35], asphalt concrete [36], and self-compacting concrete [23]. However, its application to ordinary plain concrete with relatively large coarse aggregates is scarce.

Furthermore, plain concrete specimens are usually employed in SENB fracture tests, even though reinforced concrete members are often used in actual engineering structures. The effect of steel reinforcement on concrete fracture toughness has yet to be fully explored [2].

Therefore, based on C50 ordinary concrete, which is most widely used in bridge engineering in China, this study directly compares the difference in concrete cracking performance between plain concrete and reinforced concrete specimens through three-point bending fracture tests. Longitudinal concrete strain will be recorded during the fracture test to obtain a complete load versus crack mouth opening displacement curve and a compliance curve. In addition, the crack initiation load will be determined with the help of strain gauges adhered near the prefabricated notch tip. The fracture toughness of concrete in flexural tension is obtained from test data based on traditional DKFM and more recent BEM, respectively, and the results of the two methods are compared. The obtained fracture toughness results will also be compared with those on lower-strength ordinary concrete in the literature. The novelty of this study lies in obtaining the failure pattern of C50 plain concrete specimens and an in-depth comparison of DKFM and BEM in deducing fracture toughness.

## 2. Materials and Methods

### 2.1. Materials and Mix Design

A commercial C50 grade ordinary concrete was chosen in the study due to its wide application in bridge engineering. The concrete mixture, detailed in Table 1, was designed with specific components and proportions for optimal performance. The mix ratio of the four main ingredients—cement, sand, stone, and water—was set at 1:1.93:3.02:0.46 to achieve the desired strength and workability. The choice of 42.5 Portland cement, medium natural sand with a fineness modulus of 2.4, and rubble and cobble gravel with a maximum size of 25 mm was based on their availability and suitability for bridge construction. The target slump of the fresh concrete mixture was 180 ± 20 mm, and a 1.99% high-performance water-reducing agent STD-PCS (a polycarboxylic acid-type superplasticizer manufactured by a local company, Tianjin Steady Industrial Development Co., Ltd., Tianjin, China) was added for improved workability. Additionally, 115 kg of admixtures, including mineral powder and fly ash, was used in every cubic meter of concrete to enhance its properties.

The reinforced concrete beam specimens’ reinforcement were made from the commonly used hot-rolled ribbed grade HRB400 steel bars. The steel’s properties all met standard values, including a yield strength of 400 MPa, tensile strength of 540 MPa, elastic modulus of 200 GPa, and elongation percentage of 16%, ensuring the reliability of the materials used in the study.

### 2.2. Specimen Preparation

**Figure 1:**
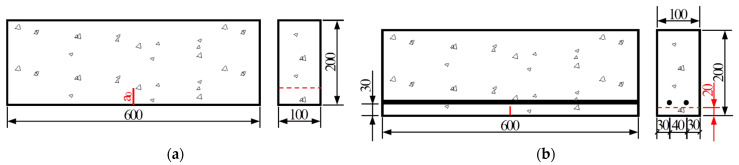
Schematic diagram of single-edge notched beam (SENB) specimens (dimensions in mm): (**a**) Plain concrete specimens (*a*_0_ varies between 20~100 mm as shown in Table 2); (**b**) Reinforced concrete specimens.

Single-edge notched beam (SENB) specimens with dimensions of 100 mm × 200 mm × 600 mm, as shown in Figure 1, were adopted for fracture test. This test was chosen to evaluate the concrete’s resistance to crack propagation, a critical factor in bridge engineering [31]. The height of the specimen, 200 mm, represents a typical lower bound value in bridge deck applications. Through-thickness straight notches of various depths were prefabricated at the midspan section of the bottom surface of SENB specimens. Altogether, 34 specimens were prepared in two series, namely, 28 for the PC series and 6 for the RC series. All PC specimens were made from plain concrete, while the RC series consisted of reinforced concrete beams. Nine companion 150 mm × 150 mm × 150 mm concrete cubes were obtained for the concrete batch to verify its grade through a compressive strength test.

For a direct comparison of crack pattern and fracture resistance, the reinforced concrete specimens were identical in geometry and material to their plain concrete counterparts except for reinforcement. A reinforcement ratio of 1% is typical in concrete bridge decks. Because the loading capacity of the test machine is limited to 100 kN (see Section 2.3 for more details) and failure of reinforced concrete is usually governed by rupture of reinforcing bars, a smaller reinforcement ratio was adopted for RC specimens. Two full-length grade HRB400 Φ8 mm reinforcing bars were placed in the bottom part of each specimen, with a concrete cover of 30 mm. A schematic diagram of the reinforced concrete specimens is shown in Figure 1b.

**Figure 2:**
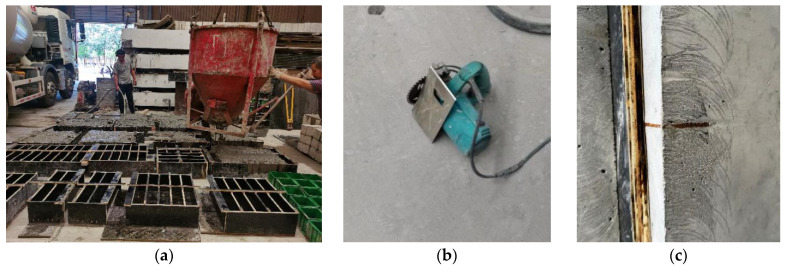
Fabrication process of the SENB specimens: (**a**) Concrete pouring; (**b**) Cutting tool for preparing shallow notches; (**c**) Embedded steel plate for preparing deeper notch.

All specimens were produced with wooden formwork. After concrete pouring, they were covered with a polyethylene sheet and cured for 28 days under standard curing conditions (at a temperature of 22 °C and a relative humidity of 95%). At the time of testing, the specimens were 30~60 days old. Figure 2a shows a photo of the specimen preparation during the concrete pouring.

The prefabricated 2 mm wide notches in SENB specimens were prepared in two manners. When the notch depth is small (notch depth-to-specimen height ratio α_0_ = 0.1), a concrete cutting machine, as shown in Figure 2b, was employed to cut the desired notch depth after concrete pouring and formwork removal. The cutting process was stopped 2~3 mm ahead of the required notch size to minimize damage to SENB specimens due to the impact of sawteeth cutting, and a positioning plate was installed to ensure uniform notch depth through specimen thickness. However, the concrete cutter cannot accomplish a deep notch. An embedded steel plate, as shown in Figure 2c of the desired dimensions, was carefully located within the formwork during concrete pouring and was taken out shortly after the initial setting of concrete to allow for the notch forming.

The 28 plain concrete specimens (PC series) are divided into 2 groups, 12 of which have a notch depth-to-specimen height ratio of 0.1 and the remaining 16 specimens have a notch-to-height ratio varying from 0.2 to 0.5. This variation in notch size was designed to obtain a compliance versus notch-to-height ratio curve from the three-point bending fracture test. The curve could be employed to deduce crack length corresponding to various loading cycles in future fatigue crack propagation tests. Six reinforced concrete specimens (RC series) were also tested for fracture performance. Details of the static fracture test specimens are shown in Table 2. The specimens were named after the Chinese Pinyin acronym. JZ stands for static loading (as opposed to fatigue loading for crack growth rate tests), W or WJ stands for plain concrete, and Y stands for reinforced concrete. The number indicates the initial notch depth in centimeters and the last digit indicates the specimen’s serial number within each group. Please note that specimens WJ-n are identical to JZ-W-2-n; however, the former were tested mainly for maximum load-carrying capacity and were not installed with strain gauges for crack initiation load measurement.

### 2.3. Testing Setups

**Figure 3:**
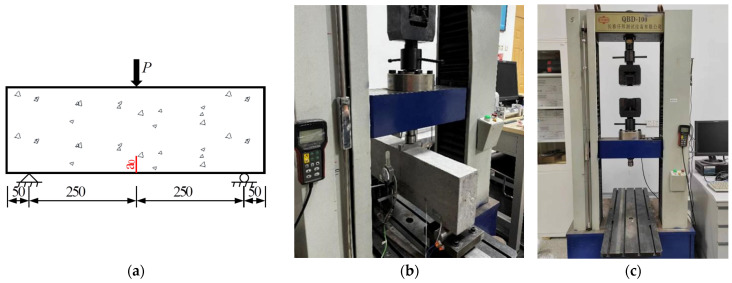
Three-point bending fracture toughness test: (**a**) Schematic drawing of test setups (dimensions in mm); (**b**) Photo of test setups; (**c**) Photo of testing machine.

All specimens were tested under a three-point bending condition and had a span-to-height ratio *S*/*H* of 2.5, as shown schematically in Figure 3a. A total of 28 specimens from the PC series and 6 specimens from the RC series were tested by a static loading test machine, as shown in Figure 3b.

The fracture test was conducted with a QBD-100 electro-hydraulic servo-controlled universal testing machine in the Engineering Mechanics Laboratory at the Beijing University of Technology. The testing machine, manufactured by Changchun Qianbang Testing Equipment Co., Ltd. (Changchun, China), has a maximum load capacity of 100 kN, as shown in Figure 3c. The loading is under displacement control, with the main transverse beam vertical movement of 0.02 mm/min. An X-Y recorder automatically recorded vertical load and transverse beam displacement.

Since static peak load will be required to determine fatigue loads for future fatigue crack propagation tests, six additional PC specimens with a notch-to-height ratio of 0.1 (specimens WJ-1 to WJ-6), in addition to JZ-W-2-n, were tested to grasp this feature more accurately. However, strain gauges were not applied for crack initiation load measurement in these six specimens.

### 2.4. Measurement and Instrumentation

**Figure 4:**
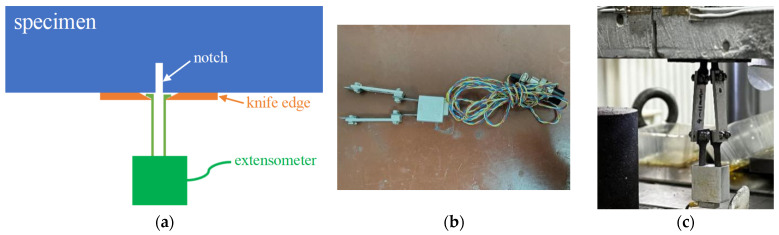
Arrangement of extensometer gauge: (**a**) Scheme of CMOD measurement; (**b**) Photo of the extensometer; (**c**) Photo of mounted extensometer.

The testing machine automatically recorded the load and displacement of the actuator, and a real-time load versus displacement curve was displayed to facilitate test monitoring. For continuous measuring of crack mouth opening displacement (CMOD) during the static test, an extensometer was mounted to the bottom surface of the specimen over a notch opening with a pair of knife edges. A YYJ-(-2)-5/6 extensometer, manufactured by NCS Testing Technology Co., Ltd. (Beijing, China), has a default gauge length of 6 mm, a measurement range between −2 mm and 5 mm, and a measuring sensitivity of 0.001 mm. Figure 4 shows the arrangement of the extensometer.

**Figure 5:**
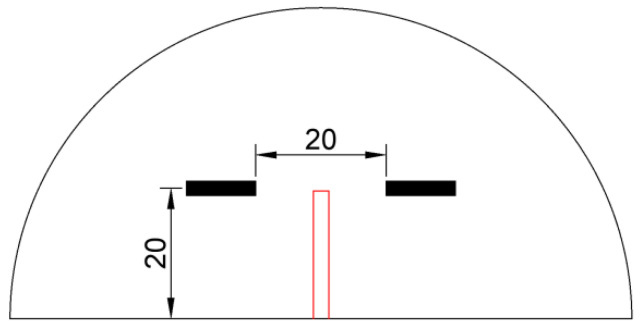
Illustration of installing strain gauges at the tip of prefabricated notches for cracking load measurement (dimensions in mm).

Resistance strain gauges were also mounted on the side surface of the specimen to monitor longitudinal strain variation in the notch tip strain field during loading. The cracking load corresponding to the initiation of concrete cracks can be determined by using reduced strain measurement. This reduction is because energy release associated with concrete cracking stops the strain value outside the crack path from increasing. Vertically, the centerline of the two strain gauges coincides with the prefabricated notch tip [37]. Longitudinally, they are symmetrical about the prefabricated notch and its extension line at 20 mm spacing. All strain gauges have a gauge length of 10 mm, width of 2 mm, and electrical resistance of 120 Ω, and the strain values were recorded with the UCS60B static data collection system. The arrangement of strain gauges for cracking load measurement is illustrated in Figure 5.

**Figure 6:**
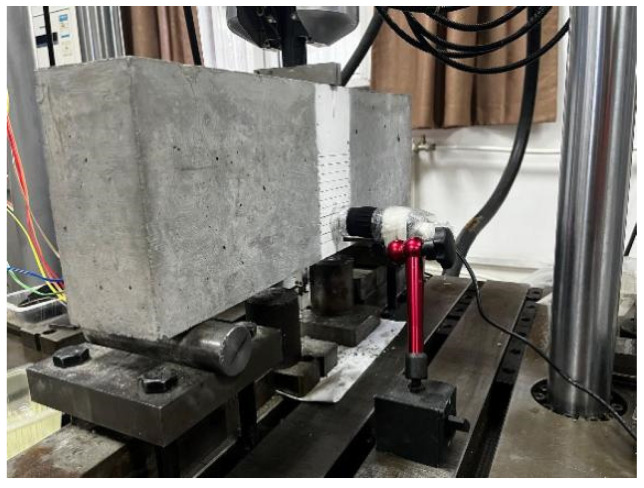
Concrete crack observation using an electronic microscope with a maximum magnification of 240×.

An electronic portable microscope MDA2000 (manufactured by Hangzhou Future Optics Sci & Tech Co., Ltd., Hangzhou, China), as shown in Figure 6, with a maximum magnification of 240×, was also used to facilitate the observation of microscopic cracks, especially when they were just initiated from the prefabricated notch. The USB digital microscope has a 2.0 MP sensor and a maximum resolution of 1600 × 1200.

### 2.5. Double-K Fracture Model

The double-K fracture model utilizes two fracture toughness values to describe the complete process of fracture. While the crack initiation fracture toughness $K_{\mathrm{IC}}^{ini}$ corresponds to cracking load *P*_ini_ and initial crack size *a*_0_ (taken as the prefabricated notch length), the unstable fracture toughness $K_{\mathrm{IC}}^{un}$ corresponds to the peak load *P*_u_ and equivalent crack size *a*_c_ just before the onset of unstable fracture. While the calculation of $K_{\mathrm{IC}}^{ini}$ is straightforward, evaluating $K_{\mathrm{IC}}^{un}$ is somewhat complicated. The difficulty lies in the determination of *a*_c_.

It is recognized that a stable crack propagation stage exists before the unstable fracture of concrete. Therefore, the actual crack size before the specimen’s unstable fracture is slightly larger than the prefabricated notch length *a*_0_. If the amount of stable crack propagation before unstable fracture, also the fictitious crack extension, is termed ∆*a*_fic_, then the equivalent crack size *a*_c_ just before the onset of unstable fracture equals *a*_0_ + ∆*a*_fic_. However, precise measurement of ∆*a*_fic_ during the experiment is complex and requires advanced measuring techniques [31]. The principle of linear asymptotic superposition has been proposed to calculate *a*_c_ [30], and $K_{\mathrm{IC}}^{un}$ can be calculated directly from peak load and the corresponding crack mouth opening displacement, eliminating the need for unloading at peak load [31,38]. For the three-point bending beam specimens with a span-to-height ratio of 2.5, the equivalent crack size *a*_c_ when the applied load reaches its peak value of *P*_u_ can be determined by Equations (1) and (2) as [31]:

(1) $$\alpha_{c}=\frac{\gamma^{3/2}+0.4460\gamma }{{\left(\gamma^{2}+2.2538\gamma^{3/2}+2.9950\gamma +3.4135\right)}^{3/4}},$$

(2) $$\gamma =CMOD_{c}\cdot B\cdot E/(6P_{u}),$$

where α_c_ = *a*_c_/*H*; *S*, *B*, *H* are the span, width, and height of the test beam, respectively; *CMOD*_c_ is the crack mouth opening displacement corresponding to *P*_u_.

Concrete’s modulus of elasticity *E* is assumed to remain constant during the loading process. It can either be measured directly from the standard test [39] or determined empirically [40] according to Equation (3):

(3) $$E=\frac{10^{5}}{2.2+(33/f_{\mathrm{cu}})},$$

where *f*_cu_ is the average strength of concrete cubes.

The equivalent fracture toughness $K_{\mathrm{IC}}^{un}$ can then be obtained by substituting *a*_c_ into Equations (4) and (5) as in [31]:

(4) $$K_{\mathrm{IC}}^{un}=\frac{3\left(P_{u}+0.5W\right)S\sqrt{\pi a_{c}}F\left(\alpha_{c}\right)}{2H^{2}B}=\frac{3P_{\max}S\sqrt{\pi a_{c}}F\left(\alpha_{c}\right)}{2H^{2}B},$$

(5) $$F\left(\alpha_{c}\right)=\frac{1.99-\alpha_{c}\left(1-\alpha_{c}\right)\left(2.15-3.93\alpha_{c}+2.7\alpha_{c}^{2}\right)}{\sqrt{\pi }\left(1+2\alpha_{c}\right){\left(1-\alpha_{c}\right)}^{3/2}},$$

where *P*_u_ is the measured peak load and *W* is the self-weight of the specimen between supports; *P*_max_ is, therefore, the modified peak load considering the effect of specimen self-weight. The cracking fracture toughness $K_{\mathrm{IC}}^{ini}$ can also be obtained from Equations (4) and (5) by replacing *P*_u_ with *P*_ini_, *a*_c_ with *a*_0_, and α_c_ with α_0_.

### 2.6. Boundary Effect Model

For quasi-brittle fracture of large structures, the nominal stress *σ*_n_ with consideration of notch length can be generally expressed as a function of the material’s tensile strength *f*_t_ and fracture toughness *K*_IC_ as [32]:

(6) $$\sigma_{n}=\frac{f_{t}}{\sqrt{1+a_{e}/a_{\mathrm{fpz}}}},$$

where *a*_e_ is the effective crack size considering boundary effect, and the scaling parameter *a*_fpz_ is the characteristic crack size determined solely by *f*_t_ and *K*_IC_ as $a_{\mathrm{fpz}}={\left(\frac{K_{\mathrm{IC}}}{1.12\cdot f_{t}}\right)}^{2}/\pi =0.25\cdot {\left(\frac{K_{\mathrm{IC}}}{f_{t}}\right)}^{2}$, symbolizing the intersection of the two asymptotic lines.

**Figure 7:**
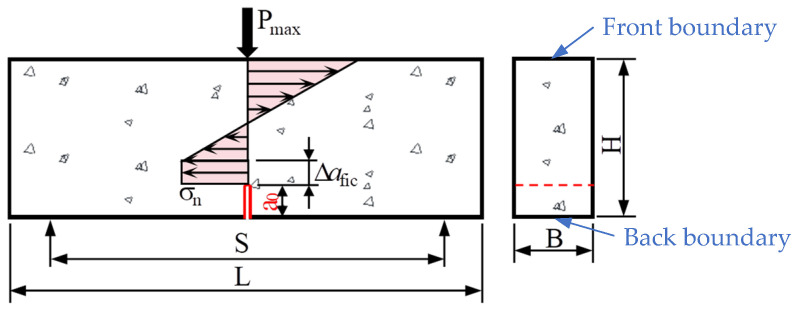
Schematic stress distribution diagram on the mid-span cross section in the boundary effect model. A fictitious crack extension ∆*a*_fic_ and a constant cohesive stress *σ*_n_ is considered.

The cohesive stress (or crack bridging stress) in FPZ can be assumed as a constant for simplicity, as shown in Figure 7. When the crack tip opening displacement at the prefabricated notch tip is small, and the fictitious crack length extension ∆*a*_fic_ is small compared to specimen height *H*, this approximation is acceptable [6,41].

Nominal stress *σ*_n_ can be derived by considering the distribution of flexural stress along the notched cross-section. Assuming a linear strain relationship, one can obtain the nominal stress *σ*_n_ that satisfies force equilibrium in the horizontal direction and equilibrium of moment as [22,33]:

(7) $$\sigma_{n}=\frac{1.5(S/B)\cdot P_{\max}}{\left(H-a_{0})(H-a_{0}+2\Delta a_{\mathrm{fic}}\right)}.$$

The effective crack size *a*_e_ for the three-point bending test, considering the effect of specimen boundaries shown in Figure 7, can be calculated by Equation (8) as [33]:

(8) $$a_{e}(\alpha_{0},a_{0})={\left[(1-\alpha_{0}{)}^{2}\cdot Y(\alpha_{0})/1.12\right]}^{2}\cdot a_{0},\alpha_{0}=a_{0}/H,$$

where shape function *Y*(α_0_) for SENB specimens with S/H = 2.5 is expressed in Equation (9) as [33]:

(9) $$Y\left(\alpha_{0}\right)=\frac{1-2.5\alpha_{0}+4.49\alpha_{0}^{2}-3.98\alpha_{0}^{3}+1.33\alpha_{0}^{4}}{{\left(1-\alpha_{0}\right)}^{3/2}}.$$

The fictitious crack length extension ∆*a*_fic_ is found to be closely related to concrete microstructure, characterized by the average diameter of coarse aggregates *d*_avg_ [33]. However, *d*_avg_ is challenging to determine for different concrete specimens [22], while each concrete mixture’s maximum coarse aggregate *d*_max_ is known in advance. Therefore, it is more convenient to use *d*_max_ as the characteristic microstructure size of concrete. Once a discrete parameter *β*_fic_ is used, the fictitious crack length extension ∆*a*_fic_ can be discretized as [23,33,36]:

(10) $$\Delta a_{\mathrm{fic}}=\beta_{\mathrm{fic}}\cdot d_{\max}.$$

Similarly, the characteristic crack size related to the fracture process zone can be expressed in terms of *d*_max_ as [33]:

(11) $$a_{\mathrm{fpz}}=0.25\cdot {\left(\frac{K_{\mathrm{IC}}}{f_{t}}\right)}^{2}=\beta_{\mathrm{ch}}\cdot d_{\max},$$

where *β*_ch_ is another discrete parameter to quantify the characteristic crack size.

Therefore, fracture toughness can be evaluated as

(12) $$K_{\mathrm{IC}}=2f_{t}\cdot \sqrt{\beta_{\mathrm{ch}}\cdot d_{\max}},$$

and concrete’s tensile strength can be obtained by combining Equations (7) and (8) as

(13) $$f_{t}=P_{\max}\cdot \frac{1.5(S/B)\cdot \sqrt{1+a_{e}/(\beta_{\mathrm{ch}}\cdot d_{\max})}}{\left(H-a_{0})(H-a_{0}+2\beta_{\mathrm{fic}}\cdot d_{\max}\right)}=P_{\max}/A_{e},$$

where the effective area $A_{e}=\frac{\left(H-a_{0})(H-a_{0}+2\beta_{\mathrm{fic}}\cdot d_{\max}\right)}{1.5(S/B)\cdot \sqrt{1+a_{e}/(\beta_{\mathrm{ch}}\cdot d_{\max})}}$ can be easily determined from the geometry and material of test specimens.

## 3. Results

### 3.1. Material Characterization

The compressive strength of the concrete mixture was obtained from standard tests on nine cubic specimens. The specimens consist of three groups, three cubes for each group, sampled randomly for concrete pouring into various formworks shown in Figure 2a. The test results and their statistical values are shown in Table 3. No significant difference was observed between the groups; as such, the results were combined. The 28-day average compressive strength of the concrete batch was 65.4 MPa, demonstrating that it meets the strength requirement of commercial-grade C50 concrete. The coefficient of variation for all nine cubes is 0.09, indicating reasonable material dispersion.

### 3.2. Failure Pattern

**Figure 8:**
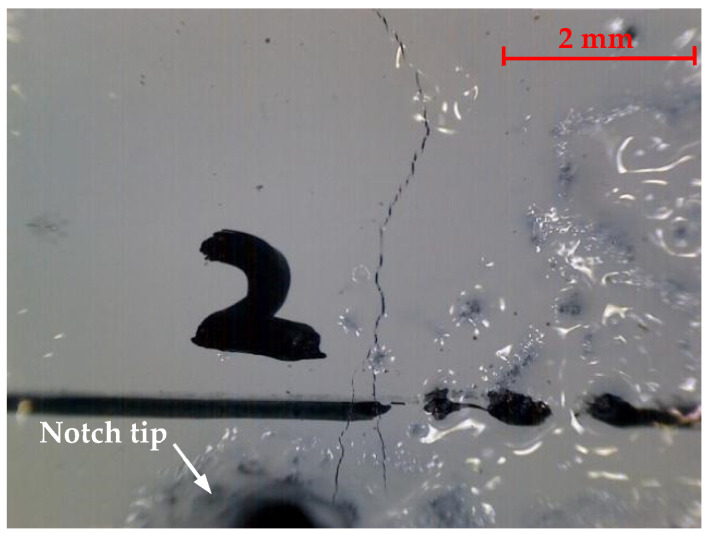
Concrete cracks observed by the electronic microscope at the tip of a prefabricated notch.

**Figure 9:**
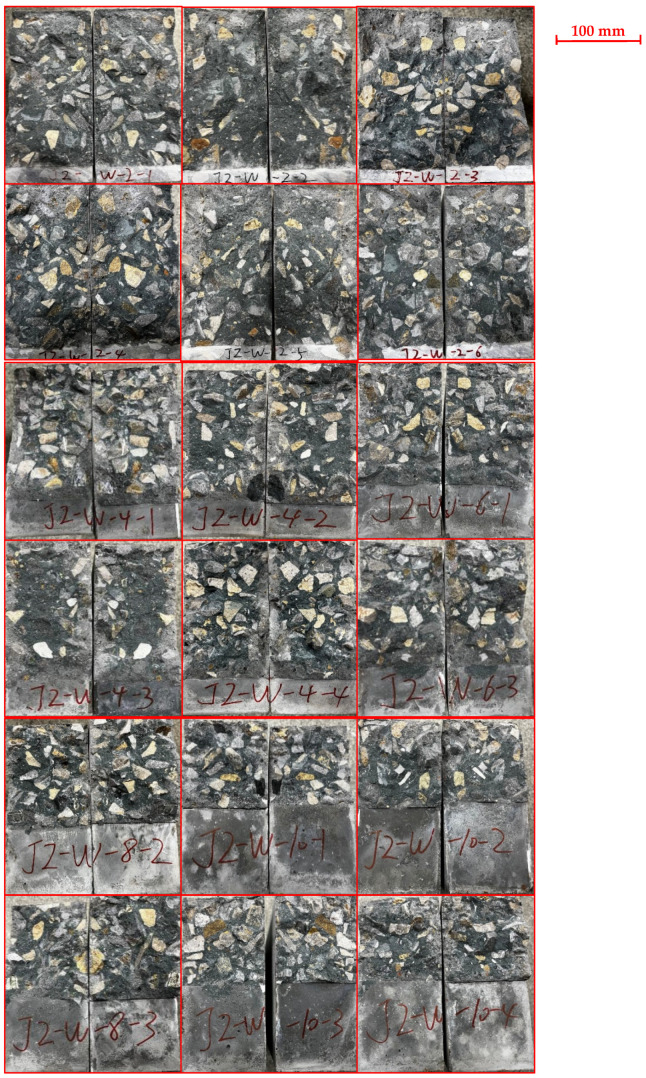
Cross-sections of the fractured plain concrete specimens. Two halves of a fractured specimen were put side by side.

**Figure 10:**
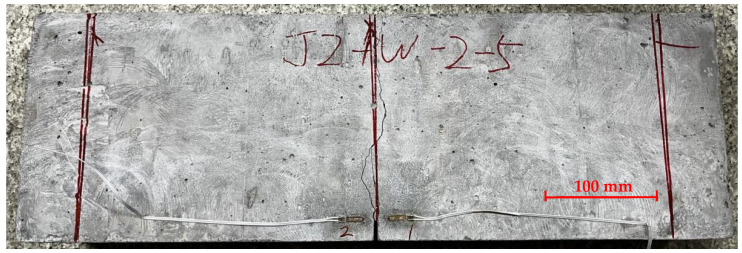
Side view of a typical fractured plain concrete specimen in which a zigzag crack is initiated from the prefabricated notch.

During the initial loading of the PC specimen, cracks were not visible to the naked eye. With the aid of the microscope, microscopic cracks initiated from the tip of the prefabricated notch of the plain concrete specimen were detected, as shown in Figure 8. Once a vertical macroscopic crack was observed to appear in mid-height, the crack developed rapidly, and the specimen fractured suddenly without much warning. All SENB specimens failed in the midspan section, and the fracture surface was generally planar. A typical failure surface of the plain concrete specimen is presented in Figure 9. Despite different lengths of prefabricated notches, the cross-sections of all failed specimens showed that about 75% of the coarse aggregates were fractured. Symmetry of coarse aggregates in both halves of a fractured surface indicates fracture of coarse aggregates. This observation means that the strength of coarse aggregates in this concrete mixture is not significantly stronger than the mortar, and cracks originating from aggregate-mortar interfaces could pass through coarse aggregates. Figure 10 shows a side view of a typical fractured plain concrete specimen. The figure shows that the crack growth pattern of the PC specimen is a vertically upward straight line along the prefabricated notch tip. Some occasionally zigzagging fracture surfaces can be explained by the pulling-out of coarse aggregates along the interface between the coarse aggregates and mortar, as shown in Figure 9.

**Figure 11:**
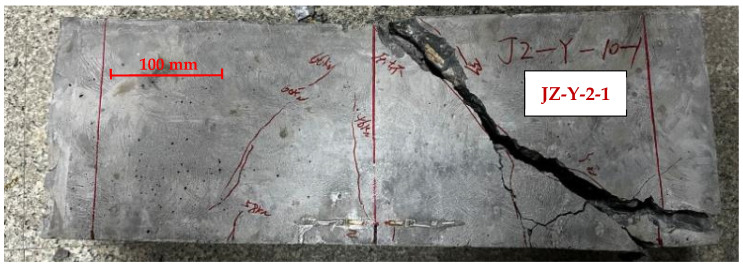
Side view of a typical fractured reinforced concrete specimen. Besides cracks initiated from the prefabricated notch, oblique cracks were also developed, eventually leading to anchoring failure. Please note that a label was added to the picture because the specimen was initially named after the notch-to-height ratio of 0.1.

For reinforced concrete specimens, small cracks also initially occurred from the prefabricated notch tip at the midspan section and appeared on the side surfaces of the concrete specimen. The crack moved upward and became macroscopic. However, the crack development did not lead to immediate failure of the specimen due to the presence of tensile reinforcement. The specimen was able to carry the increasingly applied load continuously. At the same time, oblique concrete cracks developed at support locations and slowly extended upward toward the loading point. Eventually, the test was stopped because of crushed concrete, and the load-carrying capacity of the reinforced concrete specimen was characterized by inadequate bonding and slip failure between steel reinforcement and concrete. A typical concrete failure of the reinforced concrete specimen is shown in Figure 11.

### 3.3. Crack Initiation Load

**Figure 12:**
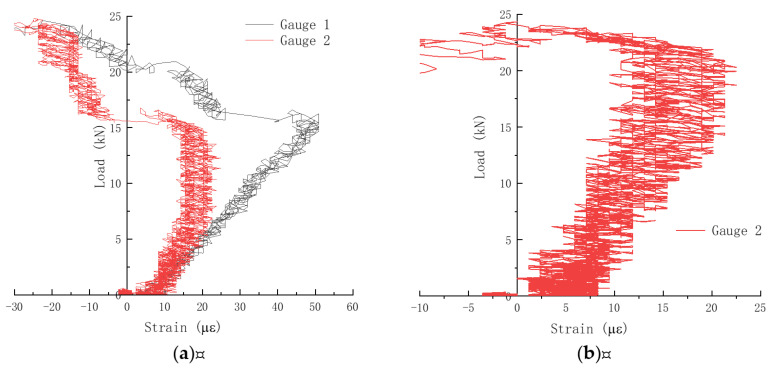
Strain measurement for determining the cracking load of plain concrete specimens at *α*_0_ = 0.1: (**a**) JZ-W-2-1; (**b**) JZ-W-2-6. Gauge 1 in JZ-W-2-6 was damaged.

**Figure 13:**
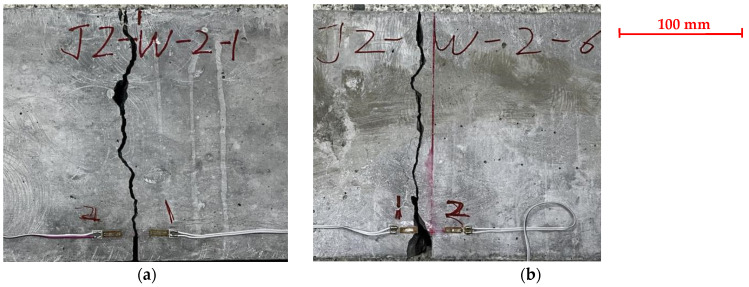
Side view of fractured plain concrete specimens at α_0_ = 0.1: (**a**) JZ-W-2-1; (**b**) JZ-W-2-6.

The cracking load beyond which cracks would initiate from the prefabricated notch tip and start to grow is determined by strain gauges affixed on both sides of the prefabricated notch. A typical strain measurement of the plain concrete specimen with a notch-to-height ratio of 0.1 is shown in Figure 12, along with a photo of the fractured specimen in Figure 13. The cracking load was determined when strain measurement stopped increasing in the load versus strain plot [42]. As seen from Figure 12a, both strain gauges in specimen JZ-W-2-1 showed reduced strain values. However, only Gauge 2 showed effective strain measurement in specimen JZ-W-2-6 as the crack grew past the location where Gauge 1 was affixed, and the strain gauge was damaged. The measured cracking load for the two specimens is 15 kN and 22.5 kN, respectively.

Measured cracking load for specimens of various notch-to-height ratios were summarized in Table 4. Because both strain gauges in specimens JZ-W-6-3 and JZ-W-8-1 were damaged during the three-pointing bending fracture test, these specimens were omitted from the table. The averaged cracking load of plain concrete specimens is 17.0 kN, 12.4 kN, 6.6 kN, 7.6 kN, and 5.3 kN with a coefficient of variation of 0.33, 0.27, 0.14, 0.26, 0.33 for notch-to-height ratios of 0.1, 0.2, 0.3, 0.4, 0.5, respectively. Generally, the cracking load decreased as the notch-to-height ratio increased.

A ratio between cracking load and peak load, *P*_ini_/*P*_u_, was also calculated where *P*_u_ was the ultimate loading capacity of the specimen when it was monotonically loaded to fracture, as explained in more detail in Section 3.4. Both cracking load and *P*_ini_/*P*_u_ ratio showed significant variations between specimens.

Similarly, the measured cracking load of the reinforced concrete specimens is shown in Table 5. The RC specimens showed an average cracking load of 20.4 kN with a coefficient of variation of 0.29. Comparing Table 5 with the values for notch-to-height ratio of 0.1 in Table 4, the measured cracking load of RC specimens is 20% higher than that of the PC specimens, and the coefficients of variation between PC and RC specimens are consistent. Reinforcement does not significantly affect concrete cracking within the concrete cover. However, the peak load of RC specimens (average value of 81.1 kN) is dramatically higher than that of PC specimens (25.6 kN), as reinforcement plays a significant role in load carrying after concrete cracking. These observations on the effect of reinforcement are consistent with those from previous investigations on tensile fatigue properties of ordinary concrete [43].

### 3.4. P-CMOD Curves

**Figure 14:**
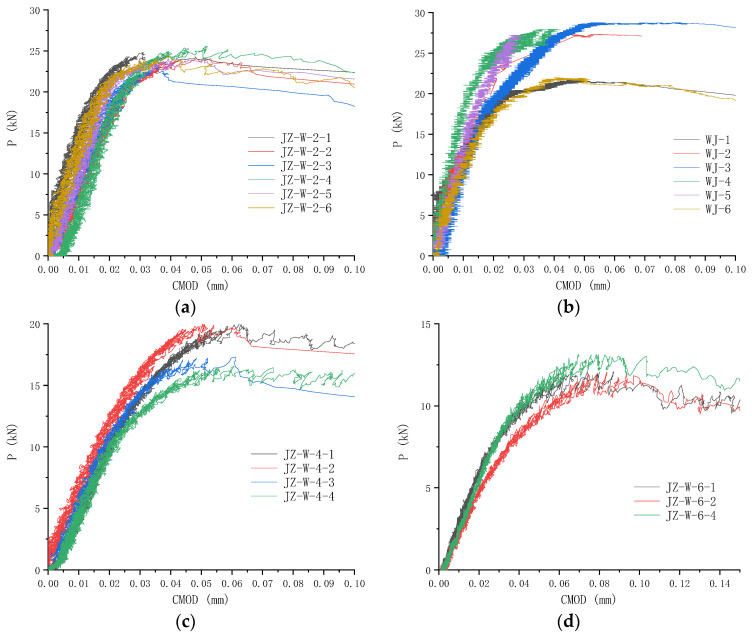

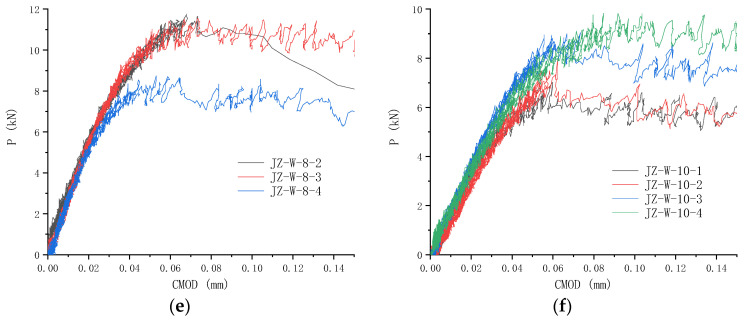
*P*-CMOD curves of plain concrete specimens with different notch-to-height ratios: (**a**) α_0_ = 0.1 (JZ-W-2-n); (**b**) α_0_ = 0.1 (WJ-n); (**c**) α_0_ = 0.2; (**d**) α_0_ = 0.3; (**e**) α_0_ = 0.4; (**f**) α_0_ = 0.5.

Load versus crack mouth opening displacement curves for PC specimens with various notch-to-height ratios are shown in Figure 14. Since no appropriate unloading devices were provided for the testing machine, the PC specimens always broke abruptly after reaching the maximum load-carrying capacity, and the measured CMOD data afterward were distorted. Therefore, Figure 14 only shows part of the *P*-CMOD curves during the loading stage up to the peak load *P*_u_. The initial straight line of load versus displacement curve demonstrates linear elastic response. As the crack initiates and propagates, the CMOD develops faster than the load, and the *P*-CMOD curve deviates.

The measured peak load and corresponding CMOD value for PC specimens with various notch-to-height ratios are listed in Table 4. As can be seen from the table, the CMOD value corresponding to *P*_u_, CMOD_c_, is relatively small, in the order of 10^−5^~10^−4^ m, and increases slightly with the increase of notch-to-height ratio. Experiments from this study also showed that once CMOD reached 0.05 mm, crack developed rapidly, and specimen fracture usually followed.

**Figure 15:**
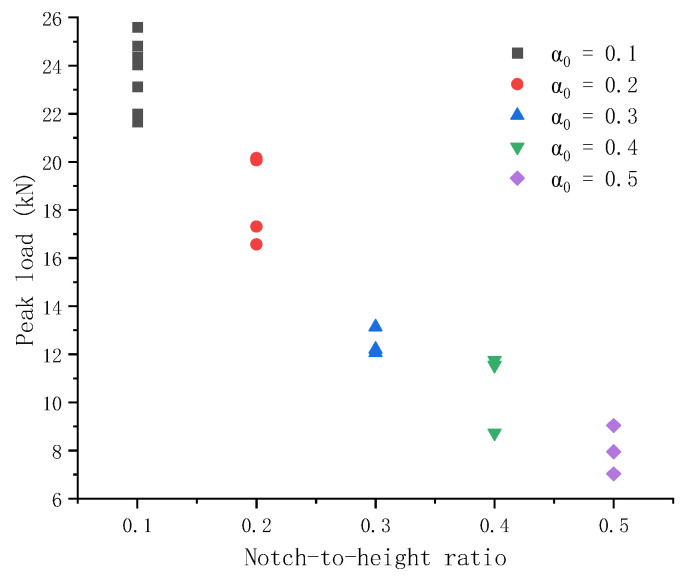
Comparison of peak load of plain concrete specimens with varied notch-to-height ratios. The peak load shows a decreasing relation with the increasing notch-to-height ratio.

Figure 15 shows the peak load of PC specimens and their notch-to-height ratio. As the notch-to-height ratio increases, the peak load that a PC specimen can sustain decreases nonlinearly.

**Figure 16:**
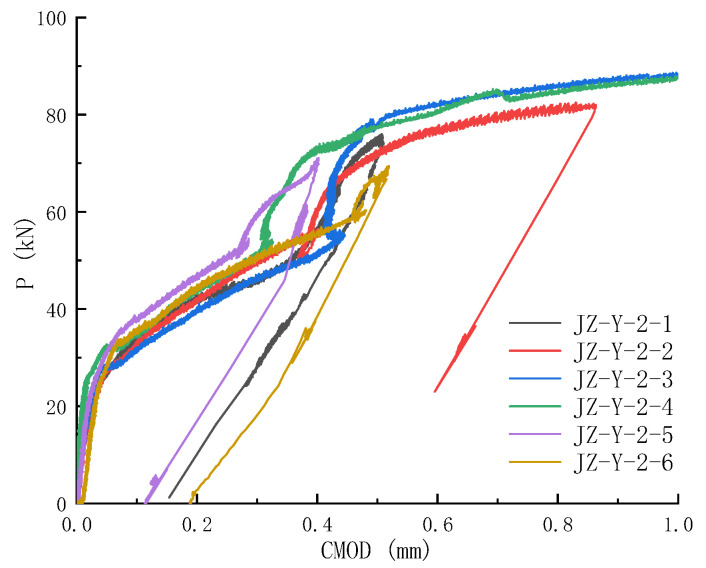
*P*-CMOD curves of reinforced concrete specimens. RC specimens show three stages of loading and have dramatically higher load-carrying capacity than their plain concrete counterparts.

The load versus crack mouth opening displacement curve for reinforced concrete specimens is similarly obtained with the aid of the extensometer and is shown in Figure 16. The loading part of the *P*-CMOD curve can be divided into three portions: the linear portion before concrete cracking, the nonlinear portion representing reinforcement action, and the last portion due to the arch action of a deep beam (with a span-to-height ratio of 2.5). The measured peak load and corresponding CMOD value for RC specimens are listed in Table 5. The load-carrying capacity of the RC specimens is 2.2 times higher than that of the PC specimens. RC specimens’ CMOD_c_ is 0.5~2 mm, at least 10 times that of PC counterparts.

### 3.5. Compliance Curve

According to Hooke’s law, stress and strain vary proportionally within the linear elastic region. A similar relation holds in this region for load *P* versus crack mouth opening displacement CMOD. The proportionality factor *C*, CMOD/*P*, is called elastic compliance, which is only related to the notch-to-height ratio as long as the geometric dimensions of the specimen and the concrete mixture are kept the same [37].

**Figure 17:**
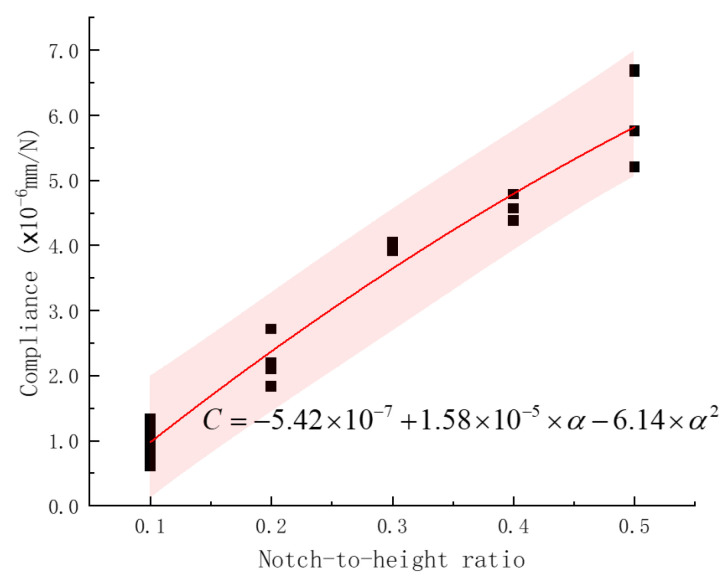
Calibrated elastic compliance versus notch-to-height ratio curve for plain concrete specimens. The compliance was defined and computed in Section 3.5. A unique notch-to-height ratio or relative crack size can be determined from one compliance value.

*P*-CMOD curves of 26 plain concrete specimens with various notch-to-height ratios were obtained and are shown in Figure 14 in Section 3.4. The elastic compliance as the slope of the linear elastic portion of each *P*-CMOD curve, obtained through linear regression analysis where CMOD was treated as the Y dataset and *P* as the X dataset, was calculated and is listed in Table 4. The compliance values are also shown in Figure 17 for different notch-to-height ratios. As the notch depth increases, the unbroken ligament decreases, and the compliance (the reciprocal of stiffness) increases. These data points, the fitted curve from regression analysis in a red line, and the 95% confidence band in pink are shown in the figure. The compliance shows a parabolic relationship with a notch-to-height ratio with an adjusted coefficient of determination of 0.96. The calibrated compliance versus notch-to-height ratio curve can be utilized to determine crack size during fatigue crack propagation tests under cyclic loading.

The elastic compliance of RC specimens was similarly obtained and is listed in Table 5. Six RC specimens’ average compliance is 9.29 × 10^−7^ mm/N with a coefficient of variation of 0.14, while that of 12 PC specimens is 9.89 × 10^−7^ mm/N with 0.23. The difference is 6.1%, indicating similar behavior between PC and RC specimens up to concrete cracking.

### 3.6. Calculated Fracture Toughness by DKFM

The method outlined in Section 2.5 does not require unloading shortly before or after the peak load is reached, which is difficult to conduct as plain concrete specimens tend to break abruptly once reaching the peak load. According to the double-K fracture model, the unstable fracture toughness $K_{\mathrm{IC}}^{un}$ can be determined from Equations (1)–(5) based on the equivalent crack size *a*_c_ just before the onset of unstable fracture. The specimen’s self-weight *W* between supports equals 0.26 kN for SENB in this study. The equivalent crack size *a*_c_ and unstable fracture toughness $K_{\mathrm{IC}}^{un}$ are calculated based on peak load *P*_u_ and CMOD_c_, and the specimen’s self-weight is considered, as listed in Table 6. Table 6 also includes the calculation of crack initiation fracture toughness $K_{\mathrm{IC}}^{ini}$.

**Figure 18:**
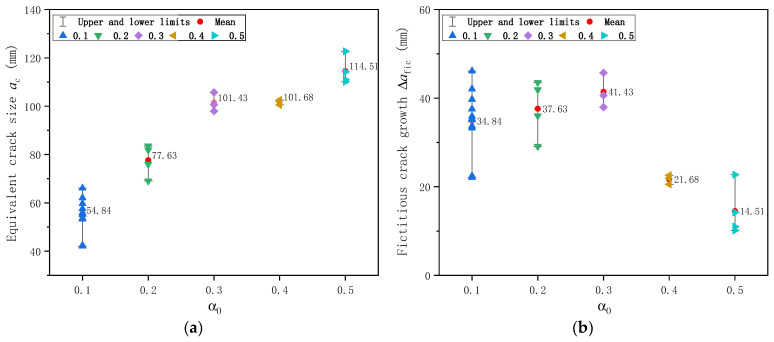
Calculated crack size based on DKFM for plain concrete specimens: (**a**) Equivalent crack size *a*_c_ just before the onset of unstable fracture; (**b**) Fictitious crack extension ∆*a*_fic_.

Based on all 26 plain concrete specimens, the calculated equivalent crack size *a*_c_ just before the onset of unstable fracture based on DKFM is shown in Figure 18a. As the initial notch size increases, the equivalent crack size *a*_c_ increases and reaches a relatively stable value after α_0_ = 0.3. The average value of equivalent crack size at fracture from these ten specimens is 106.74 mm. This calculated average stabilized value is just 6.7% higher than the experimentally observed crack size of 100 mm, in which, while the crack extends to about one-half of the specimen height, the SENB specimen tends to break very rapidly. The amount of fictitious crack extension ∆*a*_fic_, as shown in Figure 18b, increases slightly before α_0_ = 0.3 but tends to decrease after α_0_ = 0.3.

**Figure 19:**
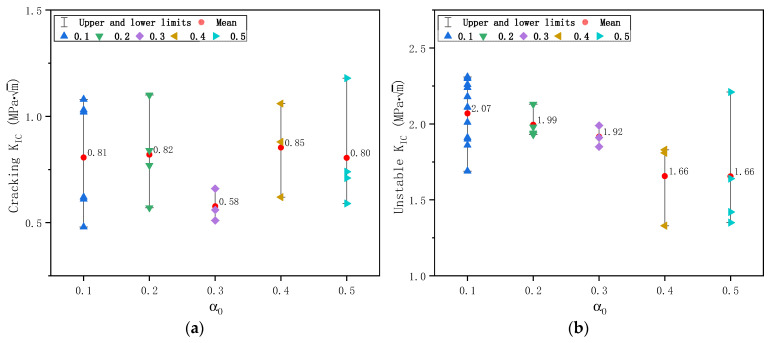
Calculated fracture toughness of plain concrete specimens based on DKFM: (**a**) Cracking fracture toughness $K_{\mathrm{IC}}^{ini}$; (**b**) Unstable fracture toughness $K_{\mathrm{IC}}^{un}$.

Similarly, the calculated fracture toughness of plain concrete specimens is shown in Figure 19 for cracking initiation fracture toughness $K_{\mathrm{IC}}^{ini}$ and unstable fracture toughness $K_{\mathrm{IC}}^{un}$. While $K_{\mathrm{IC}}^{ini}$ is unrelated to initial notch size, $K_{\mathrm{IC}}^{un}$ showed a slight dependence on initial notch size. As the initial notch size increases, $K_{\mathrm{IC}}^{un}$ decreases slightly and reaches a relatively stable value of 1.66 $MPa\cdot \sqrt{m}$. The average value, standard deviation, and coefficient of variation of $K_{\mathrm{IC}}^{ini}$ of 20 specimens determined by DKFM are 0.78 $MPa\cdot \sqrt{m}$, 0.22 $MPa\cdot \sqrt{m}$, and 0.29, and those of $K_{\mathrm{IC}}^{un}$ from 26 specimens are 1.94 $MPa\cdot \sqrt{m}$, 0.28 $MPa\cdot \sqrt{m}$, and 0.14, respectively. The scatter of $K_{\mathrm{IC}}^{ini}$ is larger than $K_{\mathrm{IC}}^{un}$, consistent with the observations in Section 3.3.

**Figure 20:**
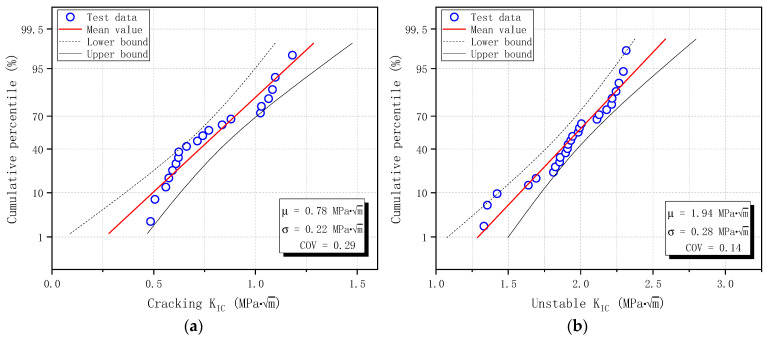
Normal distribution of fracture toughness of plain concrete specimens based on DKFM: (**a**) Cracking fracture toughness $K_{\mathrm{IC}}^{ini}$; (**b**) Unstable fracture toughness $K_{\mathrm{IC}}^{un}$.

The calculated fracture toughness is sorted in ascending order, and the Normal distribution probability plot is shown in Figure 20 for cracking initiation fracture toughness $K_{\mathrm{IC}}^{ini}$ and unstable fracture toughness $K_{\mathrm{IC}}^{un}$. The distribution parameters are estimated from input data and are shown in Figure 20, with a confidence level of 95%. The score method is Hazen, which means the cumulative percentile is calculated as (*i* − 0.5)/*n* [44], where *i* is the serial number and *n* is the total number of input data (20 for $K_{\mathrm{IC}}^{ini}$ and 26 for $K_{\mathrm{IC}}^{un}$). All test data for $K_{\mathrm{IC}}^{ini}$ and $K_{\mathrm{IC}}^{un}$ fall within the confidence band and follow the straight Normal distribution line. Therefore, the probability of measured fracture toughness based on DKFM in this study follows a Normal distribution.

### 3.7. Calculated Fracture Toughness by BEM

The optimum values of *β*_fic_ are 1.0 and 2.0 for *β*_ch_, as calibrated from experimental results [35]. Therefore, fracture toughness and tensile strength were derived according to Equations (6)–(13) for all plain concrete specimens, and the results are shown in Table 7. As mentioned above, the maximum aggregate size *d*_max_ is 25 mm, and the specimen’s self-weight between supports *W* is 0.26 kN.

**Figure 21:**
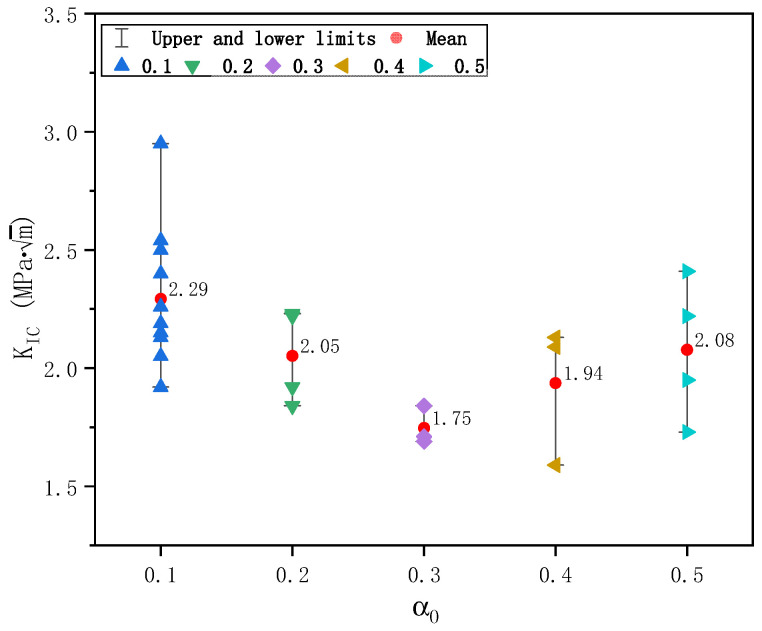
Calculated fracture toughness of plain concrete specimens based on boundary effect model.

The calculated fracture toughness *K*_IC_ based on BEM from 26 plain concrete specimens is shown in Figure 21. *K*_IC_ showed a slight dependence on initial notch size. As initial notch size increases, *K*_IC_ decreases first for α_0_ ≤ 0.3 and increases again for α_0_ ≥ 0.3. However, the difference between specimens with various initial notch sizes is insignificant. The average value, standard deviation, and coefficient of variation of *K*_IC_ of 26 specimens determined by BEM are 2.11 $MPa\cdot \sqrt{m}$, 0.30 $MPa\cdot \sqrt{m}$, and 0.14, respectively.

**Figure 22:**
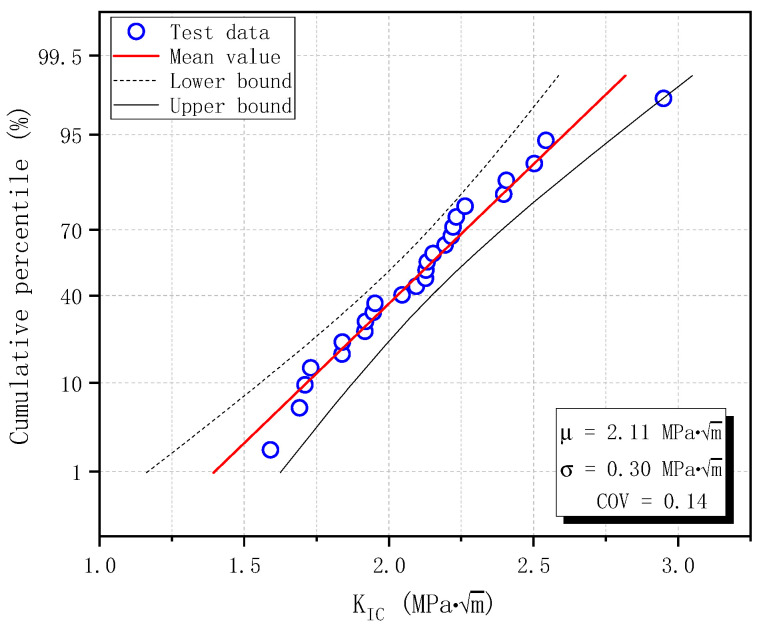
Normal distribution of fracture toughness of plain concrete specimens based on BEM.

The probability plot was obtained after arranging the calculated fracture toughness from minimum to maximum. Normal distribution is shown in Figure 22, along with the estimated distribution parameters and upper and lower bounds with a confidence level of 95%. As in Section 3.6, the cumulative percentile is calculated with the Hazen score method, which equals (*i* − 0.5)/*n* [44], where *i* is the serial number and *n* is the total number of input data (26 in this study). It is observed from the figure that all test data fall within the confidence band and follow the straight line of Normal distribution. This observation confirms that the probability of measured fracture toughness based on BEM follows a Normal distribution.

**Figure 23:**
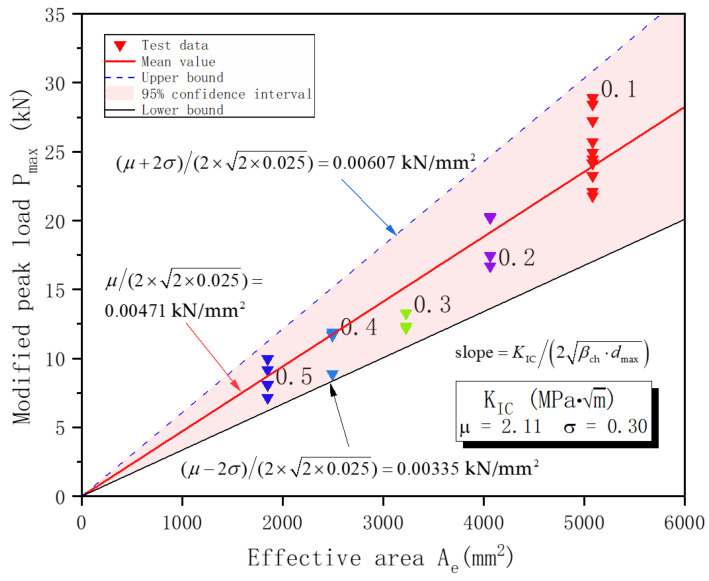
Relation between modified peak load *P*_max_ and effective area *A*_e_ based on boundary effect model. The expected value and 95% confidence band are derived for a given *A*_e_.

Utilizing the interchangeable relation between tensile strength and fracture toughness in Equation (12), the following expression $K_{\mathrm{IC}}/(2\sqrt{\beta_{\mathrm{ch}}\cdot d_{\max}})=P_{\max}/A_{e}$ is obtained by combining it with Equation (13). Please note that *P*_max_ is the modified peak load defined in Equation (4), and *A*_e_ is the effective area defined in Equation (13), solely dependent on the specimen geometry and initial notch size. Therefore, the ratio between *P*_max_ and *A*_e_ reflects the magnitude of fracture toughness, which should be a constant for a given material. Figure 23 shows all test data, plotted with *A*_e_ as the X coordinate and *P*_max_ as the Y coordinate. These data generally follow a straight line, the slope of which equals the average value of $K_{\mathrm{IC}}/(2\sqrt{\beta_{\mathrm{ch}}\cdot d_{\max}})$. Consider the inherent discreteness of material properties and fracture toughness follows a Normal distribution. The probability of data points falling within the band of (μ − 2σ, μ + 2σ), where μ and σ are the average value and the standard deviation of a Normal distribution, is 0.9544; that is, the confidence level is 95%. The upper and lower bound of *P*_max_ with a 95% confidence level can be determined from $P_{\max}=A_{e}\times (\mu \pm 2\sigma )/(2\sqrt{\beta_{\mathrm{ch}}\cdot d_{\max}})$. The estimated distribution parameters μ and σ of fracture toughness can then be utilized to predict the expected value of *P*_max_ and its upper and lower bound. It is demonstrated from the Figure 23 that all data points for specimens with different initial notch sizes fall within the confidence band.

**Figure 24:**
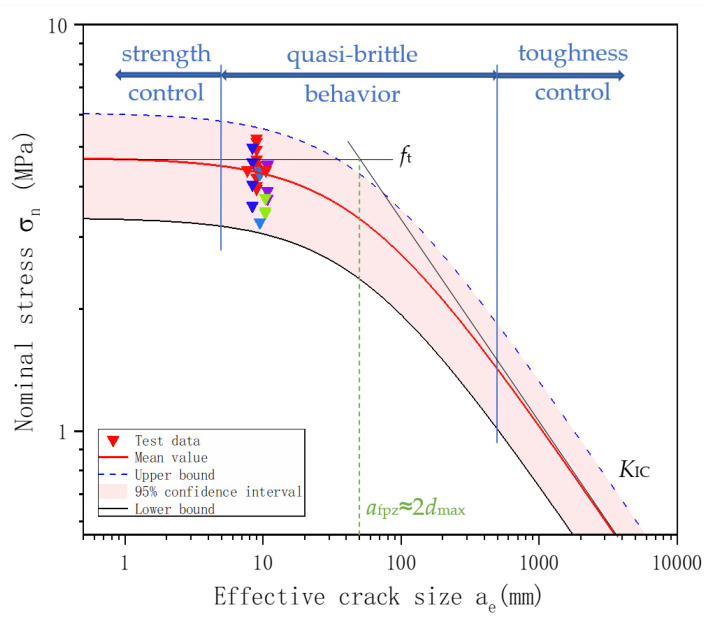
Relation between nominal stress σ_n_ and effective crack size *a*_e_. The figure demonstrates the quasi-brittle behavior of the C50 concrete in this study.

The test data are also plotted in Figure 24 for nominal stress at the crack tip σ_n_ calculated by Equation (7) against the effective crack size *a*_e_ calculated from Equations (8) and (9), considering the effect of both front and back boundaries. *a*_e_ is around 10 mm for all specimens with different initial notch sizes, between 0.1 *a*_fpz_ (5 mm) and 10 *a*_fpz_ (500 mm), indicating the quasi-brittle behavior of the C50 concrete. This behavior is expected because the specimen is relatively small, far from qualifying as the traditional plain strain fracture toughness, and strength is likely to play a more critical role. The figure also shows the estimated σ_n_ for a given *a*_e_, the mean value in a red line, and the 95% confidence band in pink. The mean value curve, the upper and lower bounds, and the two asymptotic lines are derived according to Equation (6) and utilizing statistical fracture toughness parameters. Similarly, the test results fall within the confidence band.

## 4. Discussion

### 4.1. Comparison with the Literature

Fracture toughness test data on SENB specimens under three-point bending for ordinary concrete of strength grade from 30 to 60 MPa were collected from the literature. These data and the results from this study are listed in Table 8 for a direct comparison.

The specimens from this study have the exact cross-section dimensions and span-height ratio *S*/*H* as those from the literature [29,31] but were made from concrete of different strength grades. The equivalent fracture toughness of C30 from 13 specimens is 2.37 $MPa\cdot \sqrt{m}$ [29]. As the cubic compressive strength increases, it was found that its fracture toughness decreases slightly. A 45.3~47.8% increase in strength leads to a 7.6~11.4% decrease in fracture toughness. This observation underscores the concept of fracture toughness as a quantity affected by the combined influence of strength and ductility. Technological upgrades have produced modern concrete with higher strength. However, this increase in strength is often accompanied by a decrease in ductility. If an increase in strength does not entirely compensate for the decrease in ductility, the resultant fracture toughness will decrease. Therefore, in practical engineering, close attention should be paid to the ability of higher-strength ordinary concrete to resist unstable fractures.

It is noticed from Table 8 that the maximum aggregate size from this study is higher than that from the literature [31], which might explain the decreased fracture toughness. Section 3.2 demonstrated that the proportion of coarse aggregates that fractured is about 75%. In contrast, the observation of literature reported 50% (*d*_max_ = 10 mm) for quasi-static loading with a loading rate of 0.02 mm/min [22]. The energy dissipated along the bonding interfaces between mortar and coarse aggregates as the crack propagated along the bonding interface. For the C50 concrete in this study with larger and relatively weak coarse aggregates, there is less resistance for the accumulated energy to dissipate by passing through the aggregates than developing cracks along the mortar-aggregate bonding interfaces. Therefore, cracks tended to pass through the aggregates, resulting in more coarse aggregates fractured, more straight propagation paths, and decreased ductility.

Data lines 2 and 3 in Table 8 reflect test results from beam specimens with different cross sections and span-to-height ratios made from concrete of comparable strength. The equivalent fracture toughness of C50 from 18 specimens is 1.08 $MPa\cdot \sqrt{m}$ [8], significantly smaller than that from the literature [31]. It is generally agreed that the measured concrete fracture toughness depends on the specimen geometry and dimensions [11]. Therefore, selecting the appropriate specimen type representative of the stress state in the real-world scenario of large concrete structures is crucial for a more realistic estimate of fracture toughness.

### 4.2. Comparison Between Fracture Models

Both methods are relatively straightforward in calculation. They do not require an unloading process and are also easy to implement in experiments. However, DKFM needs CMOD corresponding to the peak load, and BEM requires adopting two discrete parameters. DKFM can obtain crack initiation fracture toughness if a cracking load is available. BEM could distinguish the different failure modes by referring to effective crack size.

**Figure 25:**
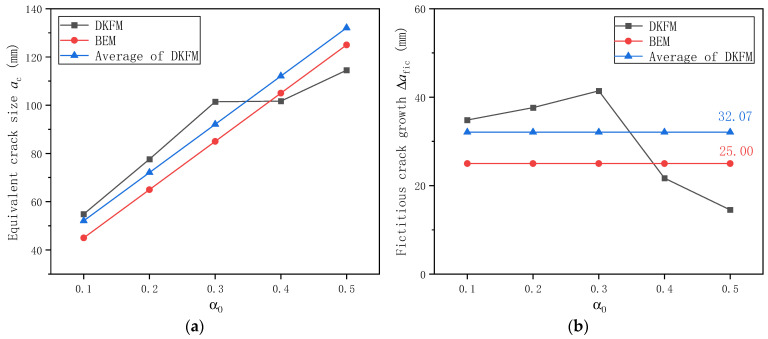
Comparison of calculated crack size for plain concrete specimens based on DKFM and BEM: (**a**) Equivalent crack size *a*_c_ just before the onset of unstable fracture; (**b**) Fictitious crack extension ∆*a*_fic_.

The equivalent crack size before fracture and the amount of crack extension are calculated for individual specimens in DKFM, as shown in Figure 18. However, a single and uniform value is assumed for all specimens in BEM, irrespective of initial notch size and variation in peak load and corresponding CMOD. For the adopted value of *β*_fic_ as 1.0 in Section 3.7 and the maximum aggregate size of 25 mm for the concrete mixture used in this study, this leads to 25 mm fictitious crack growth. Therefore, the ∆*a*_fic_ from DKFM is averaged for all specimens as 32.07 mm for comparison purposes. The calculated crack size from BEM is compared with the average value from DKFM in Figure 25 for equivalent crack size before fracture and fictitious crack extension. The result from BEM is 22.0% smaller than the average ∆*a*_fic_ calculated from DKFM for various initial notch sizes. The difference in resultant equivalent crack size *a*_c_ is 13.6%, 9.8%, 7.7%, 6.3%, and 5.4% for initial notch size from 0.1 to 0.5, respectively.

The average value, standard deviation, and coefficient of variation of unstable fracture toughness $K_{\mathrm{IC}}^{un}$ determined by DKFM are 1.94 $MPa\cdot \sqrt{m}$, 0.28 $MPa\cdot \sqrt{m}$, and 0.14, respectively, as shown in Table 6 in Section 3.6. Based on the identical 26 specimens, the average value, standard deviation, and coefficient of variation of fracture toughness *K*_IC_ determined by BEM are 2.11 $MPa\cdot \sqrt{m}$, 0.30 $MPa\cdot \sqrt{m}$, and 0.14, respectively. This comparison demonstrates that the results from the two methods are comparable: the two sets of results have the same coefficient of variation, and average values are different by less than 10% (to be specific, 8.1%). Given the extensive scatter nature inherent in concrete material properties [39,40], these are considered acceptable for engineering applications.

### 4.3. Sensitivity of β_ch_ and β_fic_

As a characteristic microstructure parameter, the maximum coarse aggregate size (*d*_max_) was adopted to represent the heterogeneity of concrete. Equations (10) and (11) show that the predicted fracture toughness depends on the two assumed discrete parameters representing the concrete discontinuity, *β*_ch_ for characteristic crack size *a*_fpz_ and *β*_fic_ for fictitious crack growth length ∆*a*_fic_, respectively. Therefore, it is necessary to analyze the sensitivity of the predicted *f*_t_ and *K*_IC_ to *β*_ch_ and *β*_fic_. ∆*a*_fic_ generally equals the *d*_max_ value for small- and medium-sized specimens, and *a*_fpz_ is generally twice *d*_max_ [35]. Besides *β*_ch_ = 2.0, *β*_ch_ = 1.0, 1.5, and 2.5 are introduced. Besides *β*_fic_ = 1.0, *β*_fic_ = 0.5, 1.5, and 2.0 are introduced. The comparison of the predicted *f*_t_ and *K*_IC_ values based on the adopted four values of *β*_ch_ and *β*_fic_ is shown in Table 9.

The coefficient of variation for *f*_t_ and *K*_IC_ are identical, as they can be calculated from each other following Equation (12). The coefficient of variation is independent of *β*_ch_ and increases slightly with an increase in *β*_fic_.

**Figure 26:**
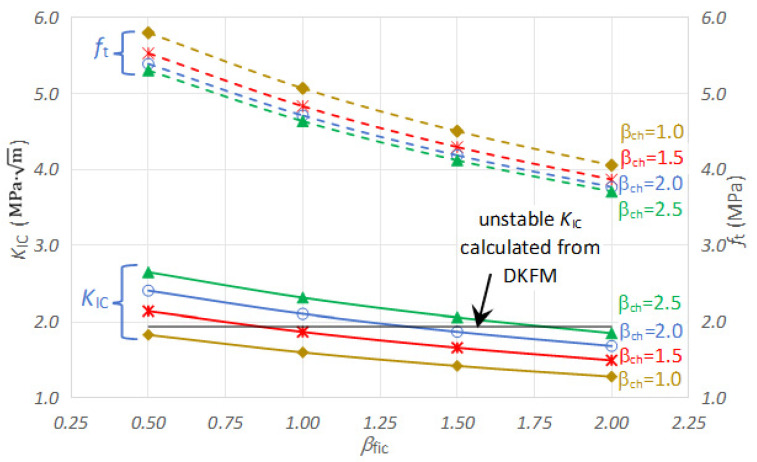
Sensitivity of discrete parameters *β*_ch_ and *β*_fic_ on the calculated average value of *f*_t_ and *K*_IC_.

Figure 26 shows the variation in mean value from Table 9 graphically, the dotted lines for *f*_t_ and solid lines for *K*_IC_. At a given *β*_ch_, *f*_t_ and *K*_IC_ decrease with the increase of *β*_fic_. The decrease rate in *f*_t_ and *K*_IC_ tends to slow down as *β*_fic_ increases. For example, for *β*_ch_ = 2.0, the decrease in *f*_t_ and *K*_IC_ is 12.5% when *β*_fic_ increases from 0.5 to 1.0; when *β*_fic_ increases from 1.5 to 2.0, the decrease in *f*_t_ and *K*_IC_ is around 9.8%. This tendency could be explained as follows. As *β*_fic_ increases, the fictitious crack growth length before fracture increases, indicating that the material is more susceptible to fracture, resulting in a decrease in *f*_t_ and *K*_IC_.

On the other hand, at a given *β*_fic_, *f*_t_ decreases with an increase of *β*_ch_, but *K*_IC_ increases with an increase of *β*_ch_. As *β*_ch_ increases, the characteristic crack size *a*_fpz_ increases, characterized by the intersection of two asymptotic lines moving toward the right side. A decrease of *f*_t_, an increase of *K*_IC_, or both could accomplish this. Equations (12) and (13) indicate that *K*_IC_ is proportional to $\sqrt{\beta_{\mathrm{ch}}}$ and *f*_t_ is proportional to $\sqrt{1/\beta_{\mathrm{ch}}}$. The magnitude of change in *K*_IC_ is more significant than that in *f*_t_. For example, for *β*_fic_ = 1.0, the decrease in *f*_t_ and increase in *K*_IC_ is 4.5% and 16.9% when *β*_ch_ increases from 1.0 to 1.5; when *β*_ch_ increase from 2.0 to 2.5, the decrease in *f*_t_ and increase in *K*_IC_ is 1.7% and 10.0%. The above analysis indicates that *K*_IC_ is slightly more sensitive to *β*_ch_ than *β*_fic_. Since the difference is less than 20%, the obtained results are not heavily dependent on selecting these discrete parameters.

Figure 26 also shows the unstable fracture toughness calculated from DKFM. It is concluded that a reasonable estimate could be obtained with appropriate combinations of *β*_ch_ and *β*_fic_. A smaller *β*_ch_ should be accompanied by a smaller *β*_fic_, and vice versa. For example, the following combinations of *β*_ch_ and *β*_fic_, expressed in data pairs of (*β*_ch_, *β*_fic_), all could lead to a close estimate of *K*_IC_ with that from DKFM: (1.0, 0.5), (1.5, 1.0), (2.0, 1.5), (2.5, 2.0).

### 4.4. Research Limitations and Future Work

It was noted in Section 3.3 that the variation in crack initiation load is much more significant than that in peak load. This relatively large variation in cracking load is probably related to notch fabrication details and the size, shape, and distribution of coarse aggregate within the specimen prefabricated notch region. Notch geometric details, especially notch tip sharpness (which can be determined from specified nominal notch radius and notch width), determine the stress concentration status of the notch tip, thus affecting the crack initiation angle and cracking load. In future experimental investigations, a consistent and acute notch tip should be specified in specimen notch preparation to obtain a more consistent crack initiation location and stable cracking load. Among the two methods of making notches, embedded steel plates are preferred over cutting machines for more accessible and practical control of the prefabricated notches. Ideally, the initial notch dimension *a*_0_ should be measured before testing if the crack initiation load is of great concern.

Fracture toughness is the primary concern in this study. Research findings on other fracture properties, such as fracture energy and critical crack tip opening displacement, should have been discussed. Although these fracture properties can be related under certain conditions, there are circumstances where other fracture properties are more relevant than fracture toughness. For example, finite element fracture analysis often requires fracture energy or critical crack tip opening displacement. Fracture energy and principal stress limit were successfully utilized to simulate the crack propagation of a plain concrete beam by the extended finite element method [45]. Further studies on plain concrete’s impermeability, frost, and corrosion resistance are also needed.

## 5. Conclusions

A three-point bending fracture test was conducted on 28 plain concrete (PC) and six reinforced concrete (RC) single-edge notched beam specimens. Based on measured strain values at the crack tip and recorded load versus crack mouth opening displacement (CMOD) curves, crack initiation load, peak load, and critical CMOD were identified. Fracture toughness was then deduced based on the double-K fracture model (DKFM) and boundary element model (BEM) and compared with test data available in the literature. The effect of reinforcement on crack initiation in RC specimens is also explored. The following conclusions are thus drawn:

(1) Crack initiation load was determined from reduced strain measurement at the crack tip. The averaged cracking load of PC specimens is 17.0 kN, 12.4 kN, 6.6 kN, 7.6 kN, and 5.3 kN with a coefficient of variation of 0.33, 0.27, 0.14, 0.26, 0.33 for notch-to-height ratios of 0.1, 0.2, 0.3, 0.4, 0.5, respectively. The scatter in crack initiation load is relatively large due to its sensitivity to local stress concentration conditions affected by notch geometric dimensions, fabrication process, and coarse aggregates around the notch tip. Notch geometry should be strictly specified, and dimensions should be measured for fracture toughness tests.

(2) The peak load, *P*_u_ of PC specimens, decreases nonlinearly with increasing notch-to-height ratios α_0_. At the same time, an inverse proportional relationship holds between the associated critical crack mouth opening displacement CMOD_c_ at peak load and α_0_ ratios. For the PC specimens with α_0_ of 0.1, *P*_u_ and CMOD_c_ mean values are 25.6 kN and 0.05 mm, respectively.

(3) Both $K_{\mathrm{IC}}^{ini}$ and $K_{\mathrm{IC}}^{un}$, indicative of crack initiation and unstable crack propagation, can be determined by DKFM. The equivalent crack size value of 107 mm agrees with experimental observation. The average value, standard deviation, and coefficient of variation of $K_{\mathrm{IC}}^{ini}$ of 20 specimens determined by DKFM are 0.78 $MPa\cdot \sqrt{m}$, 0.22 $MPa\cdot \sqrt{m}$, and 0.29, and those of $K_{\mathrm{IC}}^{un}$ from 26 specimens are 1.94 $MPa\cdot \sqrt{m}$, 0.28 $MPa\cdot \sqrt{m}$, and 0.14, respectively.

(4) BEM can predict fracture toughness with the help of measured peak load. The average value, standard deviation, and coefficient of variation of *K*_IC_ of 26 specimens determined by BEM are 2.11 $MPa\cdot \sqrt{m}$, 0.30 $MPa\cdot \sqrt{m}$, and 0.14, respectively. The two methods yield comparable results. This study verified that fracture toughness showed a Normal distribution. With an effective crack size of around 10 mm, between 0.1 *a*_fpz_ (5 mm) and 10 *a*_fpz_ (500 mm), these specimens indicate the quasi-brittle behavior of the C50 concrete.

(5) The sensitivity of the predicted *K*_IC_ to two discrete parameters, *β*_ch_ for characteristic crack size *a*_fpz_ and *β*_fic_ for fictitious crack growth length ∆*a*_fic_, was conducted. Four values were considered for each parameter. *K*_IC_ decreases with the increase of *β*_fic_ and increases with the increase of *β*_ch_. With less than 20% difference in results, BEM is not very sensitive to two assumed discrete parameters.

(6) Compared with results reported in the literature, the fracture toughness decreases slightly as the cubic compressive strength increases. A 45.3~47.8% increase in strength results in a 7.6~11.4% decrease in fracture toughness. If necessary, the experimental results in this study can be applied to other ordinary C40~C60 concrete with similar composition. However, fracture toughness tests with concrete strength grade as the sole variable parameter should be conducted to conclude the effect rigorously. As fracture toughness is affected by strength and ductility, higher-strength ordinary concrete’s ability to resist unstable fracture should be of concern.

(7) The average crack initiation load for RC specimens is 20.4 kN, 20% higher than that of PC specimens. Concrete cracking does not lead to abrupt fracture; the RC specimen continues to carry load due to reinforcement, and concrete cracks stop propagating for a significant portion of loading. The peak load in RC specimens is 81.1 kN, 2.2 times higher than in PC specimens. While PC beams generally showed crack propagation along the prefabricated notch path, the ultimate failure mode of RC beams is anchoring failure in this study. The reinforcement effect will be analyzed in more depth as more test results on RC specimens will be included.

## Figures and Tables

**Table 1:** Detailed parameters of concrete mixture, a commercial C50 concrete used in this study.

Parameters	Cement	Fine Aggregate	Coarse Aggregate	Water	Additive Agent	Mineral Powder	Fly Ash
Properties	P.O 42.5	Medium sand	Crushed, 5~25 mm	–	STD-PCS	S95	IIF
Mass per m^3^ of concrete (kg/m^3^)	347	670	1048	160	9.20	69	46
Mix ratio	1	1.93	3.02	0.46	0.03	0.20	0.13

**Table 2:** Single-edge notched beam (SENB) specimens in three-point bending fracture test. PC stands for plain concrete and RC stands for reinforced concrete.

Series	Specimen	Dimension *L* × *H* × *B* (mm)	Initial Notch Size *a*_0_ (mm)	Span/Height Ratio *S*/*H*	Notch/Height Ratio α_0_	Number of Specimens
PC	JZ-W-2-n	600 × 200 × 100	20	2.5	0.1	6
	WJ-n	600 × 200 × 100	20	2.5	0.1	6
	JZ-W-4-n	600 × 200 × 100	40	2.5	0.2	4
	JZ-W-6-n	600 × 200 × 100	60	2.5	0.3	4
	JZ-W-8-n	600 × 200 × 100	80	2.5	0.4	4
	JZ-W-10-n	600 × 200 × 100	100	2.5	0.5	4
RC	JZ-Y-2-n	600 × 200 × 100	20	2.5	0.1	6

**Table 3:** Measured 28-day compressive strength of test cubes and statistical characteristics.

Group	Measured Compressive Strength (MPa)			Statistical Characteristics		
	Cube 1	Cube 2	Cube 3	Mean Value (MPa)	Standard Deviation (MPa)	Coefficient of Variation
1	69.8	64.80	56.4	63.67	6.77	0.11
2	73.4	61.5	72.5	69.13	6.63	0.10
3	63.9	65.9	60.7	63.50	2.62	0.04

**Table 4:** Measured cracking load, peak load, CMOD_c_, and compliance for plain concrete specimens with varied notch-to-height ratios. CMOD_c_ is the crack mouth opening displacement corresponding to peak load. The elastic compliance is discussed in Section 3.5.

Specimen	Peak Load *P*_u_ (kN)	Cracking Load *P*_ini1_ (kN)	Cracking Load *P*_ini2_ (kN)	Averaged Cracking Load *P*_ini_ (kN)	*P*_ini_/*P*_u_	CMOD_c_ (mm)	Compliance (mm/N)
JZ-W-2-1	24.81	11.38	14.67	13.03	0.52	0.03	8.60 × 10^−7^
JZ-W-2-2	24.06	12.74	N.A. ^1^	12.74	0.53	0.05	1.25 × 10^−6^
JZ-W-2-3	23.12	N.A. ^1^	10.10	10.10	0.44	0.04	1.20 × 10^−6^
JZ-W-2-4	25.59	21.97	21.06	21.51	0.84	0.05	1.22 × 10^−6^
JZ-W-2-5	24.04	22.00	21.22	21.61	0.90	0.04	1.02 × 10^−6^
JZ-W-2-6	24.34	22.76	N.A. ^1^	22.76	0.94	0.04	9.16 × 10^−7^
WJ-1	21.66	N.A. ^2^	N.A. ^2^	N.A. ^2^	N.A. ^2^	0.05	1.33 × 10^−6^
WJ-2	27.11	N.A. ^2^	N.A. ^2^	N.A. ^2^	N.A. ^2^	0.05	8.56 × 10^−7^
WJ-3	28.77	N.A. ^2^	N.A. ^2^	N.A. ^2^	N.A. ^2^	0.05	8.12 × 10^−7^
WJ-4	28.31	N.A. ^2^	N.A. ^2^	N.A. ^2^	N.A. ^2^	0.05	6.07 × 10^−7^
WJ-5	33.39	N.A. ^2^	N.A. ^2^	N.A. ^2^	N.A. ^2^	0.04	7.04 × 10^−7^
WJ-6	21.99	N.A. ^2^	N.A. ^2^	N.A. ^2^	N.A. ^2^	0.06	1.10 × 10^−6^
JZ-W-4-1	20.16	11.78	11.44	11.61	0.58	0.06	2.11 × 10^−6^
JZ-W-4-2	20.06	13.78	19.38	16.58	0.83	0.05	1.83 × 10^−6^
JZ-W-4-3	17.31	8.62	N.A. ^1^	8.62	0.50	0.06	2.20 × 10^−6^
JZ-W-4-4	16.57	13.37	11.85	12.61	0.76	0.06	2.71 × 10^−6^
JZ-W-6-1	12.21	6.47	6.44	6.45	0.53	0.07	3.94 × 10^−6^
JZ-W-6-2	12.07	8.63	6.67	7.65	0.63	0.08	4.05 × 10^−6^
JZ-W-6-4	13.14	5.82	N.A. ^1^	5.82	0.44	0.07	3.91 × 10^−6^
JZ-W-8-2	11.76	6.67	8.95	7.81	0.66	0.07	4.57 × 10^−6^
JZ-W-8-3	11.55	10.15	8.82	9.48	0.82	0.07	4.38 × 10^−6^
JZ-W-8-4	8.74	4.76	6.21	5.48	0.63	0.05	4.79 × 10^−6^
JZ-W-10-1	7.03	4.85	4.84	4.85	0.69	0.06	6.70 × 10^−6^
JZ-W-10-2	7.95	4.63	4.71	4.67	0.59	0.06	6.67 × 10^−6^
JZ-W-10-3	9.04	4.34	3.36	3.85	0.43	0.07	5.21 × 10^−6^
JZ-W-10-4	9.83	6.68	8.95	7.81	0.79	0.11	5.76 × 10^−6^

^1^ Cracking load was not identified from the strain gauge. ^2^ Cracking load was not measured for specimens WJ-1 to WJ-6.

**Table 5:** Measured cracking load, peak load, CMOD_c_, and compliance for reinforced concrete specimens.

Specimen	Peak Load *P*_u_ (kN)	Cracking Load *P*_ini1_ (kN)	Cracking Load *P*_ini2_ (kN)	Averaged Cracking Load *P*_ini_ (kN)	*P*_ini_/*P*_u_	CMOD_c_ (mm)	Compliance (mm/N)
JZ-Y-2-1	76.04	13.82	N.A. *	13.82	0.18	0.51	9.05 × 10^−7^
JZ-Y-2-2	82.51	18.97	22.88	20.92	0.25	0.83	9.30 × 10^−7^
JZ-Y-2-3	93.97	25.06	24.42	24.74	0.26	2.03	1.18 × 10^−6^
JZ-Y-2-4	93.85	26.01	26.56	26.29	0.28	1.59	8.50 × 10^−7^
JZ-Y-2-5	71.04	22.30	26.06	24.18	0.34	0.40	8.25 × 10^−7^
JZ-Y-2-6	69.45	14.90	10.46	12.68	0.18	0.52	8.84 × 10^−7^

* Cracking load was not identified from the strain gauge.

**Table 6:** Crack initiation fracture toughness $K_{\mathrm{IC}}^{ini}$ and unstable fracture toughness $K_{\mathrm{IC}}^{un}$ of plain concrete specimens calculated by DKFM. $K_{\mathrm{IC}}^{ini}$ is determined from cracking load *P*_ini_ and initial crack size *a*_0_. $K_{\mathrm{IC}}^{un}$ corresponds to the peak load *P*_u_ and equivalent crack size *a*_c_ just before the onset of unstable fracture.

Specimen	*P*_ini_ (kN)	*a*_0_ (mm)	α_0_	*F*(α_0_)	$K_{\mathrm{IC}}^{ini}$ ($MPa\cdot \sqrt{m}$)	*P*_u_ (kN)	CMOD_c_ (mm)	*γ*	α_c_	*a*_c_ (mm)	*F*(α_c_)	$K_{\mathrm{IC}}^{ini}$ ($MPa\cdot \sqrt{m}$)
JZ-W-2-1	13.03	20	0.1	1.01	0.62	24.81	0.03	0.75	0.21	42.42	0.99	1.69
JZ-W-2-2	12.74	20	0.1	1.01	0.61	24.06	0.05	1.28	0.31	62.07	1.05	2.11
JZ-W-2-3	10.1	20	0.1	1.01	0.48	23.12	0.04	1.07	0.28	55.14	1.02	1.86
JZ-W-2-4	21.51	20	0.1	1.01	1.02	25.59	0.05	1.20	0.30	59.72	1.04	2.18
JZ-W-2-5	21.61	20	0.1	1.01	1.03	24.04	0.04	1.03	0.27	53.70	1.02	1.90
JZ-W-2-6	22.76	20	0.1	1.01	1.08	24.34	0.04	1.01	0.27	53.24	1.02	1.91
WJ-1	N.A. *	20	0.1	1.01	N.A. *	21.66	0.05	1.42	0.33	66.12	1.08	2.01
WJ-2	N.A. *	20	0.1	1.01	N.A. *	27.11	0.05	1.14	0.29	57.54	1.03	2.24
WJ-3	N.A. *	20	0.1	1.01	N.A. *	28.77	0.05	1.07	0.28	55.31	1.02	2.31
WJ-4	N.A. *	20	0.1	1.01	N.A. *	28.31	0.05	1.09	0.28	55.91	1.03	2.30
WJ-5	N.A. *	20	0.1	1.01	N.A. *	33.39	0.04	0.74	0.21	42.11	0.99	2.26
WJ-6	N.A. *	20	0.1	1.01	N.A. *	21.99	0.06	1.68	0.36	72.61	1.12	2.22
JZ-W-4-1	11.61	40	0.2	0.99	0.77	20.16	0.06	1.83	0.38	75.99	1.14	2.13
JZ-W-4-2	16.58	40	0.2	0.99	1.10	20.06	0.05	1.54	0.35	69.10	1.10	1.93
JZ-W-4-3	8.62	40	0.2	0.99	0.57	17.31	0.06	2.14	0.41	81.88	1.20	1.98
JZ-W-4-4	12.61	40	0.2	0.99	0.84	16.57	0.06	2.23	0.42	83.55	1.21	1.94
JZ-W-6-1	6.45	60	0.3	1.04	0.56	12.21	0.07	3.53	0.50	100.61	1.43	1.85
JZ-W-6-2	7.65	60	0.3	1.04	0.66	12.07	0.08	4.08	0.53	105.70	1.51	1.99
JZ-W-6-4	5.82	60	0.3	1.04	0.51	13.14	0.07	3.28	0.49	97.97	1.39	1.91
JZ-W-8-2	7.81	80	0.4	1.18	0.88	11.76	0.07	3.67	0.51	101.94	1.45	1.83
JZ-W-8-3	9.48	80	0.4	1.18	1.06	11.55	0.07	3.74	0.51	102.58	1.46	1.81
JZ-W-8-4	5.48	80	0.4	1.18	0.62	8.74	0.05	3.53	0.50	100.53	1.42	1.33
JZ-W-10-1	4.85	100	0.5	1.42	0.74	7.03	0.06	5.26	0.57	114.20	1.68	1.35
JZ-W-10-2	4.67	100	0.5	1.42	0.71	7.95	0.06	4.65	0.55	110.13	1.60	1.42
JZ-W-10-3	3.85	100	0.5	1.42	0.59	9.04	0.07	4.77	0.55	110.99	1.61	1.64
JZ-W-10-4	7.81	100	0.5	1.42	1.18	9.83	0.11	6.90	0.61	122.71	1.91	2.21

* Cracking load was not measured for specimens WJ-1 to WJ-6.

**Table 7:** Fracture toughness *K*_IC_ of plain concrete specimens calculated by boundary effect model. The discrete parameters, *β*_fic_ for fictitious crack extension and *β*_ch_ for characteristic crack size, are taken as 1.0 and 2.0, respectively.

Specimen	Peak Load *P*_u_ (kN)	Initial Notch Size *a*_0_ (mm)	α_0_	Y(α_0_)	*a*_e_ (mm)	*f*_t_ (MPa)	$K_{\mathrm{IC}}$ ($MPa\cdot \sqrt{m}$)	σ_n_ (MPa)	*a*_e_/*a*_fpz_
JZ-W-2-1	24.81	20	0.1	0.93	8.98	4.91	2.19	4.52	0.18
JZ-W-2-2	24.06	20	0.1	0.93	8.98	4.76	2.13	4.38	0.18
JZ-W-2-3	23.12	20	0.1	0.93	8.98	4.57	2.05	4.21	0.18
JZ-W-2-4	25.59	20	0.1	0.93	8.98	5.06	2.26	4.66	0.18
JZ-W-2-5	24.04	20	0.1	0.93	8.98	4.76	2.13	4.38	0.18
JZ-W-2-6	24.34	20	0.1	0.93	8.98	4.81	2.15	4.43	0.18
WJ-1	21.66	20	0.1	0.93	8.98	4.29	1.92	3.95	0.18
WJ-2	27.11	20	0.1	0.93	8.98	5.36	2.40	4.93	0.18
WJ-3	28.77	20	0.1	0.93	8.98	5.69	2.54	5.24	0.18
WJ-4	28.31	20	0.1	0.93	8.98	5.60	2.50	5.15	0.18
WJ-5	33.39	20	0.1	0.93	8.98	6.60	2.95	6.07	0.18
WJ-6	21.99	20	0.1	0.93	8.98	4.35	1.95	4.01	0.18
JZ-W-4-1	20.16	40	0.2	0.91	10.77	4.99	2.23	4.53	0.22
JZ-W-4-2	20.06	40	0.2	0.91	10.77	4.97	2.22	4.51	0.22
JZ-W-4-3	17.31	40	0.2	0.91	10.77	4.29	1.92	3.89	0.22
JZ-W-4-4	16.57	40	0.2	0.91	10.77	4.11	1.84	3.73	0.22
JZ-W-6-1	12.21	60	0.3	0.95	10.40	3.82	1.71	3.48	0.21
JZ-W-6-2	12.07	60	0.3	0.95	10.40	3.78	1.69	3.44	0.21
JZ-W-6-4	13.14	60	0.3	0.95	10.40	4.11	1.84	3.74	0.21
JZ-W-8-2	11.76	80	0.4	1.07	9.48	4.77	2.13	4.37	0.19
JZ-W-8-3	11.55	80	0.4	1.07	9.48	4.68	2.09	4.29	0.19
JZ-W-8-4	8.74	80	0.4	1.07	9.48	3.56	1.59	3.26	0.19
JZ-W-10-1	7.03	100	0.5	1.30	8.37	3.87	1.73	3.58	0.17
JZ-W-10-2	7.95	100	0.5	1.30	8.37	4.36	1.95	4.04	0.17
JZ-W-10-3	9.04	100	0.5	1.30	8.37	4.95	2.22	4.59	0.17
JZ-W-10-4	9.83	100	0.5	1.30	8.37	5.38	2.41	4.98	0.17

**Table 8:** Comparison of *K*_IC_ from this study with data from the literature for ordinary plain concrete of strength grade from 30 to 60 MPa. Only fracture toughness on SENB specimens under three-point bending was compared.

Reference	*f*_cu_ (MPa)	Water/Cement Ratio	*d*_max_ (mm)	Dimension *L* × *H* × *B* (mm)	*S*/*H*	Number of Datapoints	α_0_	$K_{\mathrm{IC}}^{ini}$ ($MPa\cdot \sqrt{m}$)	$K_{\mathrm{IC}}^{un}$ or $K_{\mathrm{IC}}$ ($MPa\cdot \sqrt{m}$)	Analytical Method
[29]	30.4	0.52	N.A. *	550 × 200 × 100	2.5	13	0.20~0.50	N.A. *	2.37	DKFM
[31]	44.9	0.52	10	550 × 200 × 100	2.5	15	0.20~0.50	1.03	2.07	DKFM
[8]	51.2	0.27	20	650 × 150 × 150	4.0	18	0.40~0.55	N.A. *	1.08	N.A. *
Current study	65.4	0.46	25	600 × 200 × 100	2.5	20 or 26	0.10~0.50	0.78 N.A. *	1.94 2.11	DKFM BEM

* Not reported in the literature.

**Table 9:** Sensitivity of discrete parameters, *β*_ch_ for characteristic crack size and *β*_fic_ for fictitious crack extension, on calculated tensile strength *f*_t_ and fracture toughness *K*_IC_.

*β*_ch_	*β*_fic_	*f*_t_			*K*_IC_		
		Mean Value (MPa)	Standard Deviation (MPa)	Coefficient of Variation	Mean Value ($MPa\cdot \sqrt{m}$)	Standard Deviation ($MPa\cdot \sqrt{m}$)	Coefficient of Variation
1.0	0.5	5.79	0.79	0.14	1.83	0.25	0.14
1.0	1.0	5.06	0.72	0.14	1.60	0.23	0.14
1.0	1.5	4.50	0.68	0.15	1.42	0.21	0.15
1.0	2.0	4.05	0.64	0.16	1.28	0.20	0.16
1.5	0.5	5.52	0.76	0.14	2.14	0.30	0.14
1.5	1.0	4.83	0.70	0.14	1.87	0.27	0.14
1.5	1.5	4.29	0.65	0.15	1.66	0.25	0.15
1.5	2.0	3.87	0.61	0.16	1.50	0.24	0.16
2.0	0.5	5.39	0.75	0.14	2.41	0.33	0.14
2.0	1.0	4.71	0.68	0.14	2.11	0.30	0.14
2.0	1.5	4.19	0.64	0.15	1.87	0.28	0.15
2.0	2.0	3.77	0.60	0.16	1.69	0.27	0.16
2.5	0.5	5.30	0.74	0.14	2.65	0.37	0.14
2.5	1.0	4.63	0.67	0.14	2.32	0.34	0.14
2.5	1.5	4.12	0.63	0.15	2.06	0.31	0.15
2.5	2.0	3.71	0.59	0.16	1.85	0.30	0.16

## Data Availability

Details of the experimental data presented in this study are available upon reasonable request to the corresponding author. The data are not publicly available due to privacy.
